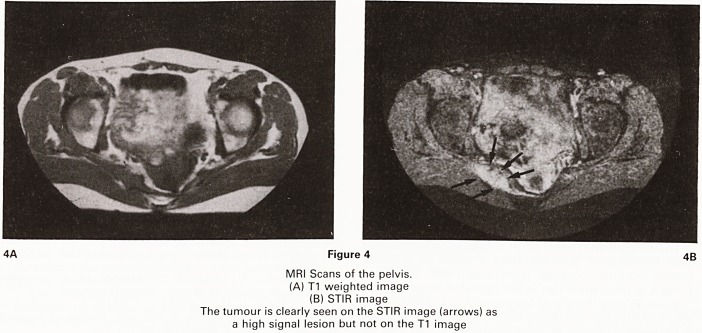# The Detection of Recurrent and Metastatic Malignant Disease in the Pelvis Using MRI

**Published:** 1988-05

**Authors:** P. Goddard, W. Wong, R. Yeats, A. Case, J. Tawn, J. Browning, E. Whipp


Bristol Medico-Chirurgical Journal Volume 103 (ii) May 1988

### The Detection of Recurrent and Metastatic Malignant Disease in the Pelvis using MRI

**P. Goddard MD FRCR, W. Wong MBBS, R. Yeats, A. Case, J. Tawn, J. Browning FRCS MRCOG, E. Whipp FRCR**

### INTRODUCTION

Magnetic resonance imaging has been successfully used to demonstrate a variety of primary diseases in the pelvis. There has been a particular emphasis on benign and malignant gynaecological tumours (1–3). MRI has considerable potential in the detection of recurrent and metastatic malignant disease of all kinds of the pelvis in both male and female patients.

Fifteen patients with previously treated primary malignant disease of the pelvis were investigated in order to determine the presence or absence of recurrent or metastatic disease. The patients were scanned used a Picker 2055 Hp 0.5 T MRI scanner and also had computerised tomography and ultrasound scans.

| | | | |
|---|---|---|---|
| Patients: | male | | 6 |
| | female | | 9 |
| Primary Diagnoses: | carcinoma | bladder | 3 |
| | | cervix | 6 |
| | | prostate | 1 |
| | | rectum | 2 |
| | lymphoma | | 1 |
| | teratoma | | 1 |
| | chondrosarcoma | | 1 |

MR sequences used included T1 weighted scans in the sagittal and transverse plane (Spin echo TR 500 msec TE 26 msec) and a short tau inversion recovery (STIR) sequence (TR 1500 msec TI 100 ms).

The T1 sequences were excellent for demonstrating the anatomy and for showing metastatic disease in bone.

On the STIR sequences malignant lesions were all shown as areas of high signal intensity (white) and this provided high contrast with soft tissue.

Greater detail or extent was shown on MR in 6 of the 15 patients. The new findings included better delineation of the primary tumour in three patients, bone metastases in two and soft tissue metastatic disease in one case.

The following two case reports illustrate some of the ways in which MR scanning is useful in the management of recurrent malignant disease in the pelvis.

**Case Report 1**

A seventy-six year old man presented with a two month history of macroscopic haematuria. I.V.P. and cystoscopy revealed an extensive transitional carcinoma chiefly involving the left side of the bladder and also the right side at the base and the anterior wall. Histological examination showed a grade T1 transitional carcinoma but a clinical classification of grade T3 was made. The patient had a course of radical radiotherapy to the bladder.

Two months after completion of treatment the patient developed dysuria and frequency of micturation. Cystoscopic examination revealed some residual ulceration which was calcified but otherwise showed a satisfactory regression of the tumour. Two small bladder stones were removed by biopsy forceps. Five months later two small local recurrences were removed cystoscopically.

Three months after that during a routine follow-up cystoscopic examination extensive recurrence at the bladder neck was discovered. An urgent CT scan of the pelvis was performed to assess suitability for cystectomy. Unfortunately streak artefacts originating from a left hip prosthesis prevented satisfactory images from being obtained. (Figure 1).

An MRI scan of the pelvis was performed. It demonstrated the tumour clearly confined to the bladder wall, with no invasion to perivesical fat. (Figure 2)

A total cystectomy was performed. Histological examination confirmed the localised extent of the tumour.

**Figure 1**

CT Scan of pelvis. The image is degraded by linear artefacts produced by the left hip prosthesis

**Figure 2**

MRI Scan of the pelvis. T1 weighted image. The tumour is clearly identified within the bladder wall (arrow)

**Case Report 2**

A fifty-one year old woman had an abdominoperineal resection for a rectal carcinoma. Histological examination of the resected bowel indicated a poorly differentiated tumour with infiltration to the surrounding fat and involvement of lymph nodes (Dukes' C).

Ten months later she developed a pain over her sacrum and around the perineum.

One month after that she began to experience difficulty in initiating micturition and occasional urge incontinence. A CT scan of the pelvis was performed. The uterus was found to be retroverted into the space normally occupied by the rectum but no evidence of recurrent tumour was present. (Figure 3A).

An ultrasound examination of the pelvis was also performed. It confirmed the presence of a retroverted uterus but no tumour was evident. (Figure 3B).

Two months later an MRI scan of the pelvis was performed. T1 and T2 weighted images did not demonstrate any recurrent tumour. STIR sequences however revealed a high signal lesion in the muscle wall of the right hemipelvis posteriorly, strongly suggestive of local tumour recurrence. (Figure 4A, B). External radiotherapy treatment was given. Her symptoms were abolished completely.

She remained well when she was seen three months later.

**Acknowledgement**

This work would not have been possible without the support of the Dawn James Trust and the Bristol MRI Scanner Fund.

**REFERENCES**

1. MAWHINNEY, R. R. POWELL, M.C. WORTHINGTON, B. S. SYMONDS, E. M. (1988) "Magnetic Resonance Imaging of Benign Ovarian Masses" *BJR* **61**, 179–186
2. DOOMS, G. C. HRICAK, H. TSCHOLAKOFF, D. (1986) "Adnexal structures: MR imaging". *Radiology* **158**, 639–646.
3. POWELL, M. SYMONDS, E. M. WORTHINGTON, B. S. (1986) "The application of MRI to gynaecology". *British Journal of Hospital Medicine* **35**, 393–403.

3A

**Figure 3** 3B

(A) CT Scan of pelvis.
(B) Longtitudinal Ultrasound Scan of pelvis in the plane of the bladder. No evidence of tumour is seen.
The uterus is retroverted into the site of the removed rectum

4A **Figure 4** 4B

MRI Scans of the pelvis.
(A) T1 weighted image
(B) STIR image
The tumour is clearly seen on the STIR image (arrows) as a high signal lesion but not on the T1 image

## Figures and Tables

**Figure 1 f1:**
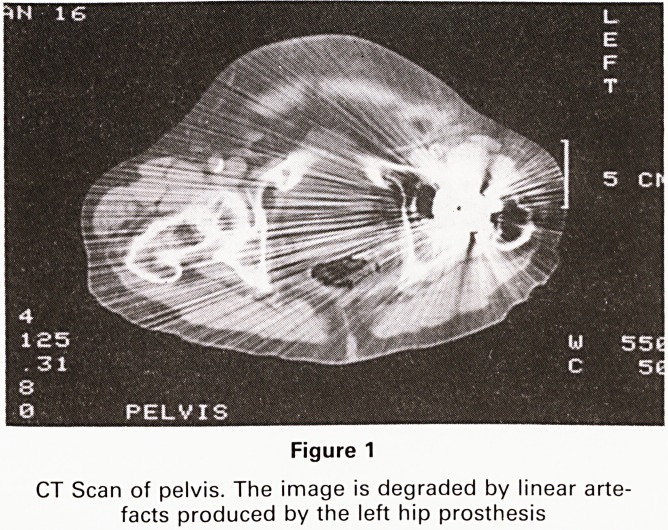


**Figure 2 f2:**
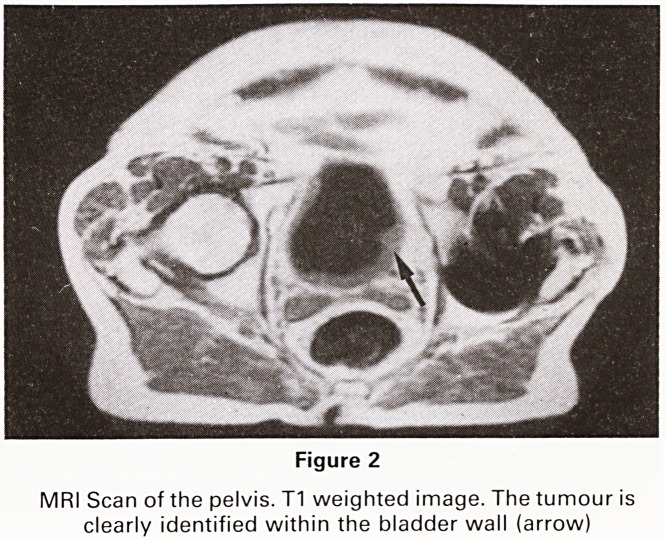


**Figure 3 f3:**
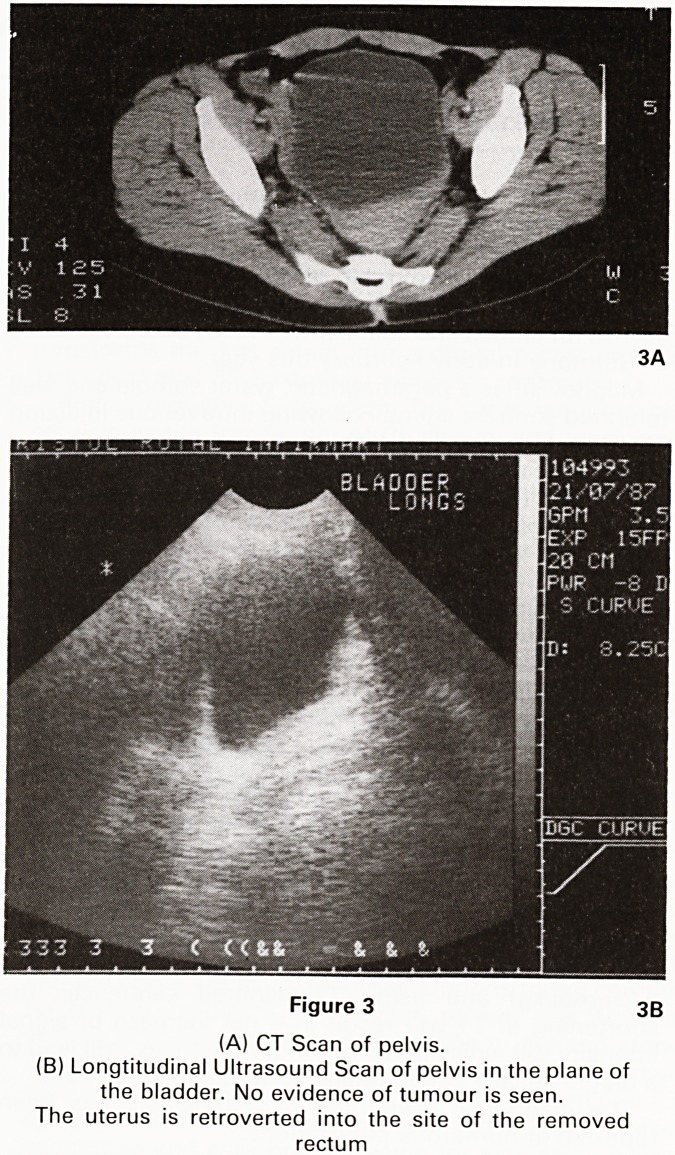


**Figure 4 f4:**